# Late Cenozoic History of the Genus *Micromys* (Mammalia, Rodentia) in Central Europe

**DOI:** 10.1371/journal.pone.0062498

**Published:** 2013-05-06

**Authors:** Ivan Horáček, Markéta Knitlová, Jan Wagner, László Kordos, Adam Nadachowski

**Affiliations:** 1 Department of Zoology, Charles University, Praha, Czech Republic; 2 Institute of Geology AS CR, v.v.i., Praha, Czech Republic; 3 Magyar Földtani és Geofizikai Intézet, Budapest, Hungary; 4 Institute of Systematics and Evolution of Animals PAS, Krakow, Poland; Monash University, Australia

## Abstract

Molecular phylogeography suggests that *Micromys minutus*, the sole extant species of the genus, colonized its extensive range quite recently, during the Late Pleistocene-Holocene period. Rich Pliocene and Pleistocene fossil records both from Europe and China suggest rather continuous and gradual *in situ* phenotype rearrangements from the Pliocene to the Recent periods. To elucidate the discrepancy we reexamined a considerable part of the European fossil record of the genus (14 sites from MN15 to Q3, 0.4–4.2 Ma, including the type series of *M. preaminutus* from MN15 Csarnóta 2), analyzed them with the aid of detailed morphometric comparisons, and concluded that: (a) The European Pliocene form, *M. praeminutus,* differs significantly from the extant species; (b) it exhibits a broad phenotypic variation covering the presumptive diagnostic characters of MN16 *M. caesaris*; (c) despite having smaller dimensions, the Early and Middle Pleistocene forms (MN17-Q3, 2.6–0.4 Ma) seem to be closer to *M. praeminutus* than to the extant species; (d) the extinction of *M. praeminutus* during Q3 and the re-occupation of its niche by the recent expansion of *M. minutus* from E-European – C Asiatic sources (suggested by phylogeographic hypotheses) cannot be excluded. Discussing interpretations of the phylogenetic past of the genus we emphasize the distinct history of the West Palearctic clade (Late Miocene-Early Pleistocene) terminating with *M. praeminutus* and the East Asiatic clade (*chalceus, tedfordi, minutus*), and the possible identity of the Western clade with the Late Miocene genus *Parapodemus.*

## Introduction

Compared to other clades of the largest mammalian family of Muridae, the genus *Micromys* shows several striking peculiarities. The genus is composed of a single extant species, *Micromys minutus* (Pallas, 1771), one of the smallest forms of the family. Although it resembles the synchorological genera *Apodemus* or *Mus* (tribes Apodemini and Murini *sensu* [1]) in its dental and cranial phenotype, robust molecular evidence demonstrated that it is a sister clade of the genus *Rattus* s.l., and belongs thus to the basal branch of the family, tribe Rattini ([1]–[3]). The extant species occupies a giant panpalearctic range, from England and France to the Far East, SE China, Taiwan and Japan. Against expectation, the molecular phylogeography ([4]) revealed that the extant species is a homogeneous entity without a trace of cryptic species variation and exhibiting no deeper phylogeographic substructures: European and East Asian samples showed a net nucleotide divergence of only 0.36%, suggesting the initial divergence time of these population to be some 80 ka B.P. only.

The last point is particularly striking because the genus is almost continuously represented in both Chinese and European fossil record since the latest Miocene. Specifically, the genus is present in almost all European assemblages of the Pliocene age (MN15-16, 2.5–4.2 Ma) and can even be looked upon as an index taxon of that period (comp. [5]–[6]). This is particularly pertinent for the Mediterranean region, where it exhibited considerable taxonomic diversity reflected by subsequent descriptions of 7 fossil species ([7]–[10]). Most of the European Pliocene records are co-identified with the Late Ruscinian taxon *Micromys praeminutus* Kretzoi, 1959 (type locality MN15 Csarnóta 2, ca 4 Ma), diagnosed as the form resembling the extant species in most characters but differing by having significantly larger dimensions [11]. The respective fossil taxon was intended to cover an earlier grade of the extant form, and alternatively, the separate species status of *Micromys praeminutus* was doubted at least for the Late Pliocene and Quaternary records of *Micromys*, which were often coidentified directly with the Recent species ([12]–[16]). The phenotypic characteristics of the sparse Pleistocene records of *Micromys* in Europe seem to support the existence of gradual phenotypic shifts interconnecting the extant species and the somewhat larger Pliocene form (cf. [13]–[15], [17]–[18]).

Obvious discrepancies between the paleobiogeographic scenario suggested by molecular phylogeography (see above) and the picture provided by the fossil record call for a detailed re-examination of the topic.

In these connections it should be remembered that the fossil record of the genus is actually rather sparse. Although *Micromys* is reported from a relatively large number of European fossil assemblages (7 MN13–14, 18 MN15–16, 7 MN17, 8 Q1–Q2, 3 Q3 sites), in most instances the material is restricted to just a few isolated teeth as a rule, often only a single one. Very few localities provided the larger series necessary for understanding patterns of variation, and in only a few of these were these variations actually examined (see Dataset S1 for details). Our study is centered on one such series obtained from an early MN17 site, Včeláre 6, in SE Slovakia ([19]–[20]), i.e. from the time period critical for re-examining the hypothesis on the gradual transition between MN15 *M. preaminutus* and extant *M. minutus*. Unfortunately, the original description of *M. praeminutus* lacks any detail, and the meaning of the taxon as it appears in secondary literature and/or in differential diagnoses of further fossil species is actually not based on the phenotype characteristics of the type series but on items chosen *ad hoc* from other localities (Sète, Moreno 2, Limni, Węże etc.). Naturally, our study begins with a re-examination of the variation pattern in the type series of *M. praeminutus* and a discussion of its actual content and relations to both the extant form and named fossil species.

## Materials and Methods

We examined 103 items identified as *Micromys* spp. (mostly isolated teeth and jaw fragments) from 14 Pliocene and Early Pleistocene sites in Central Europe. Namely, these were:

**MN15 Csarnóta 2** (Hungary, coll. MAFI Budapest): 8 M1, 13 m1 (type series of *Micromys praeminutus* Kretzoi, 1959; see [11], [21] for details of the site).

**MN15 Gundersheim-Findling** (Germany, coll. SMF Frankfurt a.M.): 2M1,2m1 [22]. **MN15 Vinogradovka 2** (Ukraine, casts in coll. SMF Frankfurt a.M.): 3 m1, 1 m2 (see [23]). **MN15 Vinogradovka 3** (Ukraine, casts in coll. SMF Frankfurt a.M.): 1 m1 (see [23]). **MN16 Ręmbielice-Królewskie II** (Poland, coll. ISEZ PAN Krakow): 4M1, 2M2, 4 m1, 1m2, 1m3 (see [24]). **MN16 Ręmbielice-Królewskie I** (Poland, coll. ISEZ PAN Krakow): 1M1, 2m1, 1 m2 ([24]). **MN16 Zhevakhova Gora 15** (Ukraina, casts in coll. SMF Frankfurt a.M.): 3 M1, 4 m1, 3 m2 [23]. **MN 16/17 Gundersheim** – Heller’s collection (Germany, coll. SMF Frankfurt a.M.): 1M1, 1m2 [25], [22]. **MN17 Včeláre 6/1** (Slovakia, coll. UK Praha): 13 maxilary fragment, 10 mandibular fragments, 12 M1, 8 M2, 4M3, 9m1, 9 m2, 4 m3 (see [19] and [26] for details of the site). **MN17 Zamkowa Dolna Cave** (Poland, coll. ISEZ PAN Krakow): 2 m1 (see [27]). **MN17 Kotlovina 3** (Ukraine, casts in coll. SMF Frankfurt a.M.) : 1 M1, 2 m1 (see [23]). **MN17 Beremend 11** (Hungary, coll. HMNH Budapest): 1 M1, 1 m1, 1 m2 (see [28]). **Q2 Hohensülzen** (Germany, coll. SMF Frankfurt a.M.): 2 m1 (see [13] for details).

In addition, we examined the type series of *Micromys chalceus* Storch, 1987 from the Chinese MN13 site Ertemte 2∶18 M1, 6 M2, 12 m1, 8 m2, 1 m3 (casts in coll. SMF Frankfurt a.M.; see [29] for details) and a comparative series of 64 skull and mandibles of extant *Micromys minutus* from the Czech Republic and Slovakia (coll. Dept. Zoology, Charles University Prague), as well as 13 items from the Holocene/Vistulian sites, Rejtek and Tarkö (both Hungary) and Soutěska II, Zazděná, and Bašta (all Czech Republic) – comp. [20]. The examination of the materials deposited in the above mentioned institutions in Czech Republic, Hungary and Poland was undertaken as part of research projects at the respective institutions (the authors - IH,LK,AN are ultimate authorities responsible for the respective collections). The material deposited in Senckenberg Institute, Frankfurt a.M. (SMF), concerned the previously published items (see Dataset S1) and for purpose of the present project were examined by JW under permission of the collection curator (K. Krohmann).

**Figure 1:**
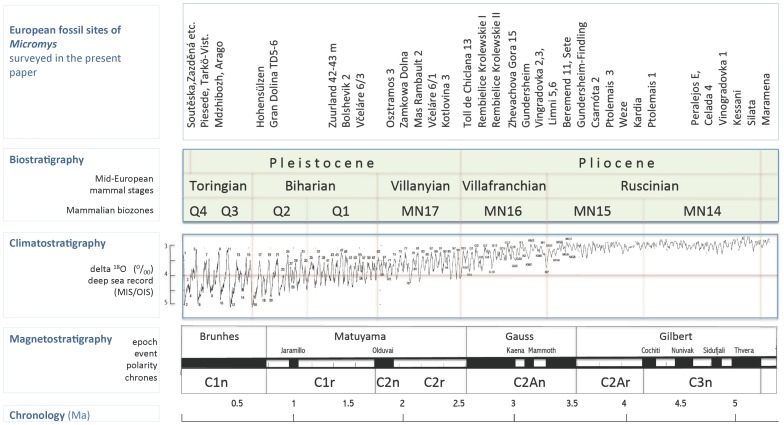
Stratigraphic context of the European Pliocene and Pleistocene records of *Micromys*. For a detailed list of sites see Dataset S1. For details concerning standard biostratigraphic subdivisions see e.g. [20]–[21], [30]–[34], the climatostratigraphic curve compiled after global benthic delta ^18^O record surveyed by [76], magnetostratigraphic standards and chronology after [77].

The stratigraphical position of particular samples is expressed in terms of the standard European biostratigraphic scale ( [30], [32]) and alternatively in units of the Neogene (MN) or Quaternary (Q) mammalian biozones ([31], [20],[33]–[34]). For further stratigraphic correlates see Fig. 1.

**Figure 2:**
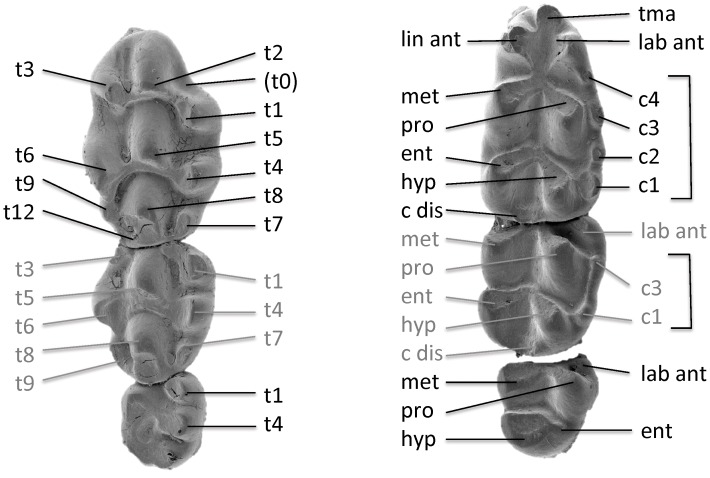
Cusp nomenclature of murid molars applied in the present paper.

We follow the cusp nomenclature proposed by van de Weerd [35] and Storch [29] with regards to alternatives suggested by Lazzari et al. [36] – cf. Fig. 2. The mandibular molars are denoted here in lower case (m1–3), the maxillar molars in upper case (M1–3).

**Figure 3:**
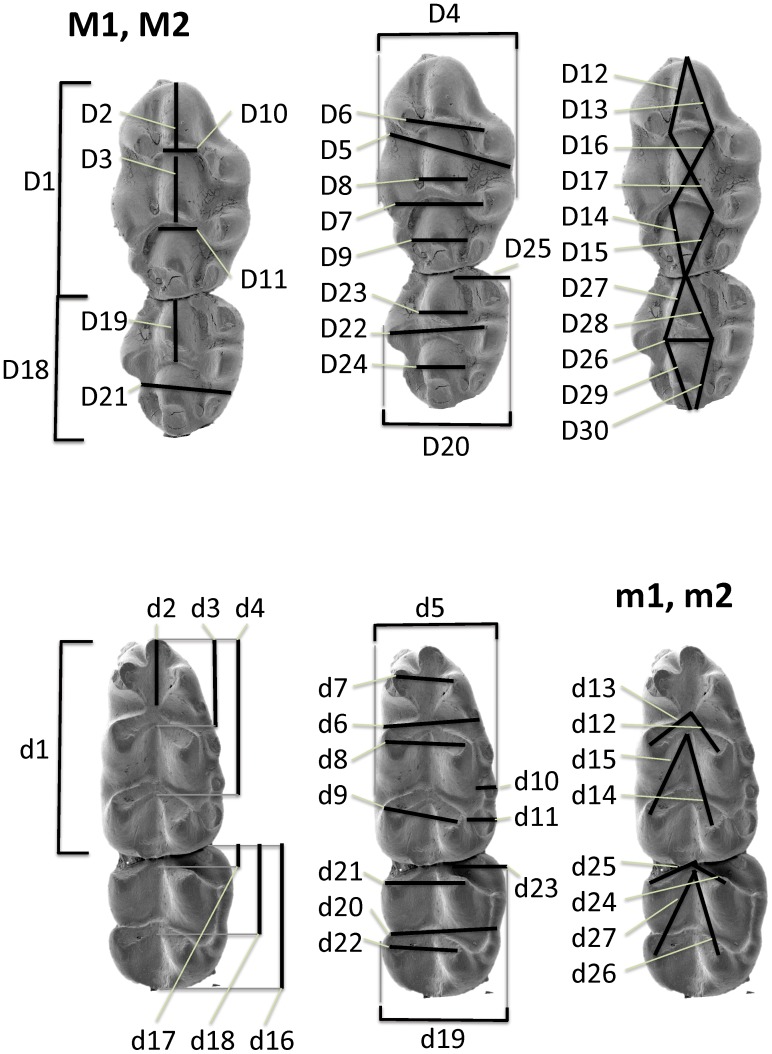
Graphical outline of the metric variables used in the present paper.

**Figure 4:**
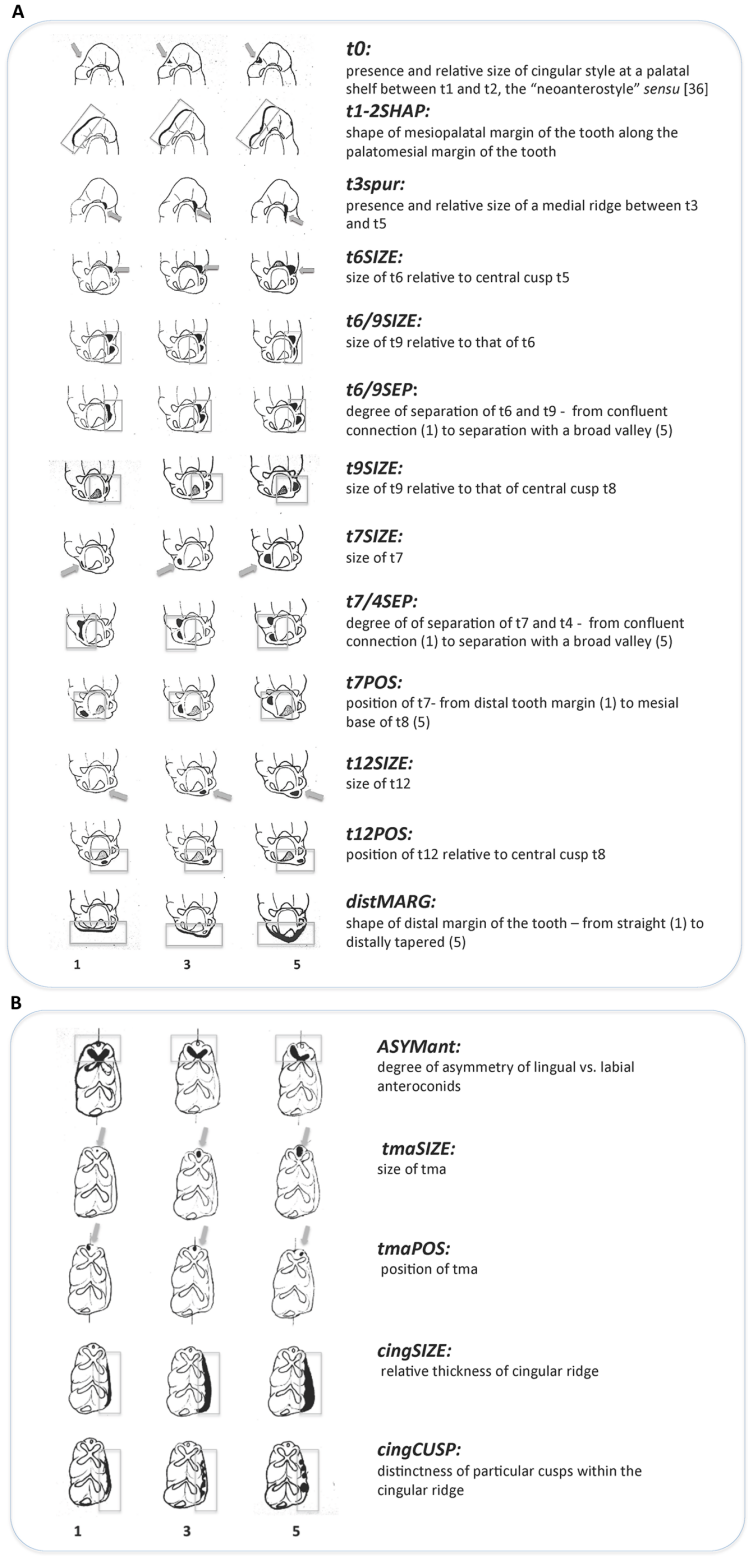
Graphical outline of the non-metric variables used in the present paper and character states 1,3,5 illustrating the calibration scales (1–5) applied in scoring the non-metrical dental characters. A. Graphical outline of the non-metric variables of M1 used in the present paper and character states 1,3,5 illustrating the calibration scales (1–5) applied in scoring the non-metrical dental characters. B. Graphical outline of the non-metric variables of m1 used in the present paper and character states 1,3,5 illustrating the calibration scales (1–5) applied in scoring the non-metrical dental characters.

**Figure 10:**
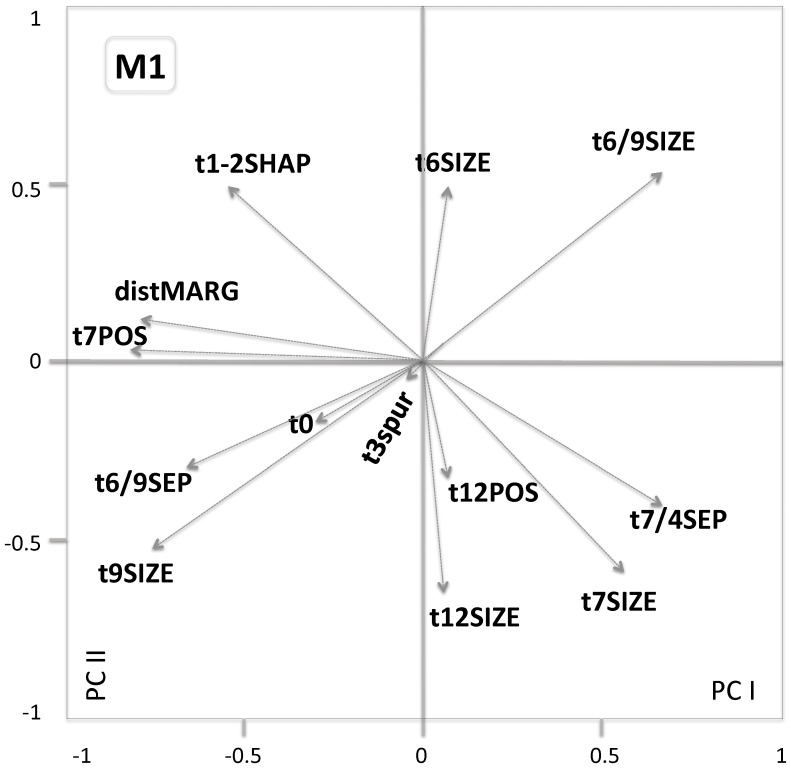
PCA factor scores (PC1,2) of particular M1 variables (in total sample) – note dominant role and mutual independence of components of t9 and t7 complexes.

**Figure 11:**
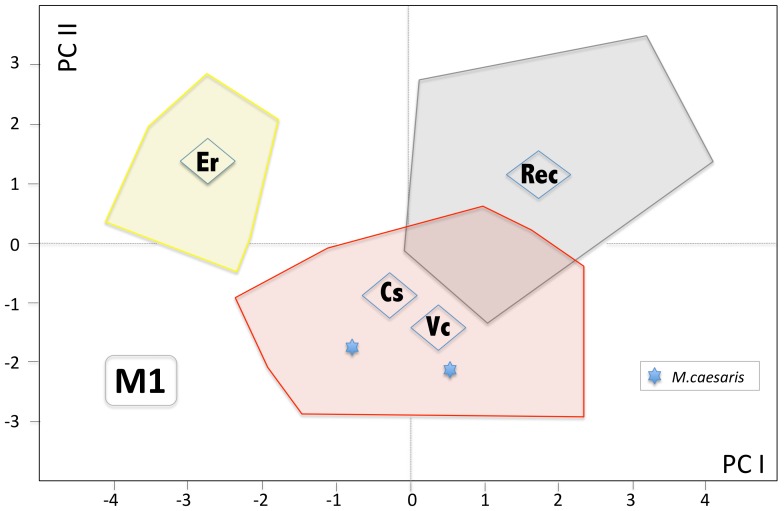
Scatter plot of PC1 and PC2 factor loadings of PCA of non-metrical M1 variables (varimax normalized) with visualized clouds of the Recent sample (*M. minutus* Czech Republic - Rec) - grey, *M. chalceus* (Ertemte - Er) – yellow, and the European Pliocene and Early Pleistocene populations – red, with position of respective centroids (Cs-Csarnóta 2, Vc-Včeláre 6/1) and type and paratype of *Micromys caesaris* (scored based on the published SEM figures in [10]).

All items were photographed (mostly using an OLYMPUS ZX27 stereomicroscope). 56 dental measurements were taken (see Fig. 3) with the aid of tpsDig image analysis software (by F.J. Rohlf). The states of 18 non-metrical variables were scored in terms of predefined character-specific scales (0,1.5) covering the observed variation; their definitions are in Fig. 4. Statistical analyses were performed using Statistica Software 8. Exploration analyses (correlation matrices, ANOVA, principal components analyses) were computed both for the set of all variables and, for metric and non-metric variables separately. The PCA results shown in Fig. 10–11 refers to set of non-metric variables.

## Results

**Figure 5:**
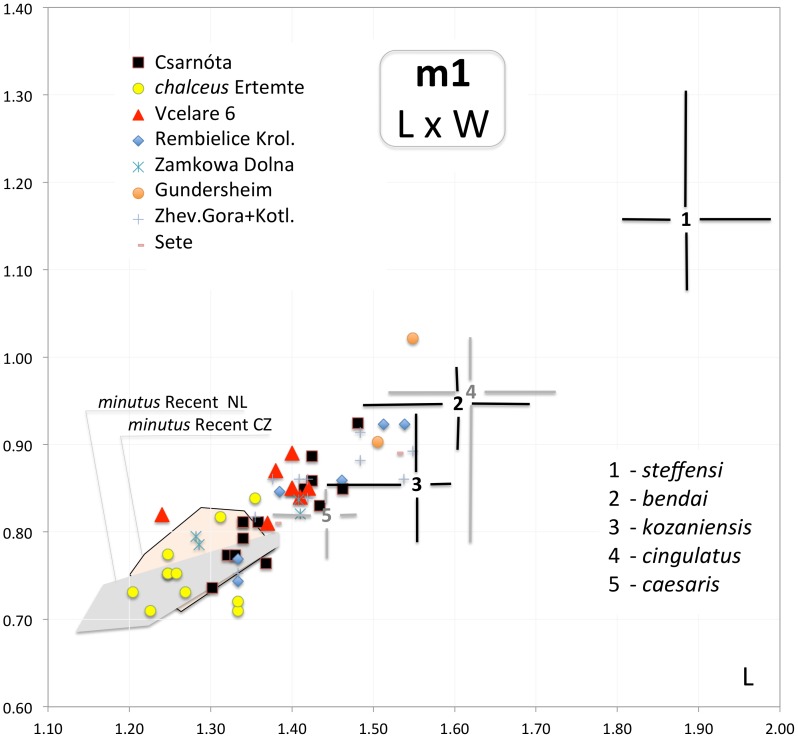
Scatter plot of m1 length (d1) and m1 width (d4) of particular items of fossil *Micromys* compared to variation span in selected fossil taxa (based on literary sources) and Recent *Micromys minutus* from Czech Republic (Recent CZ) and the Netherlands (Recent NL – after [8]). Data from Sète after [12].

**Figure 6:**
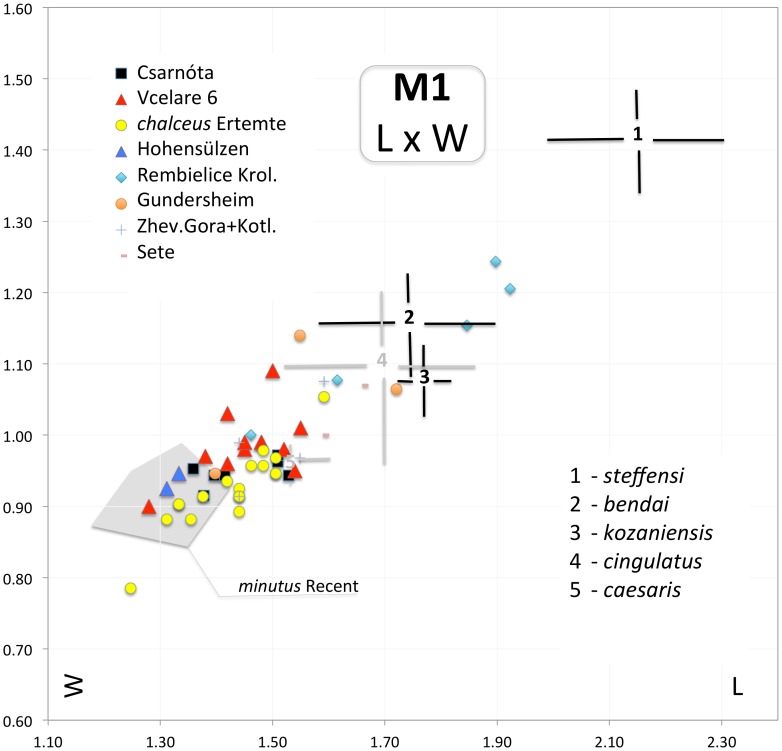
Scatter plot of M1 length (D1) and M1 width (D5) of particular items of fossil *Micromys* compared to variation span in selected fossil taxa (based on literary sources) and Recent *Micromys minutus* from Czech Republic. Data from Sète after [12].

**Figure 7:**
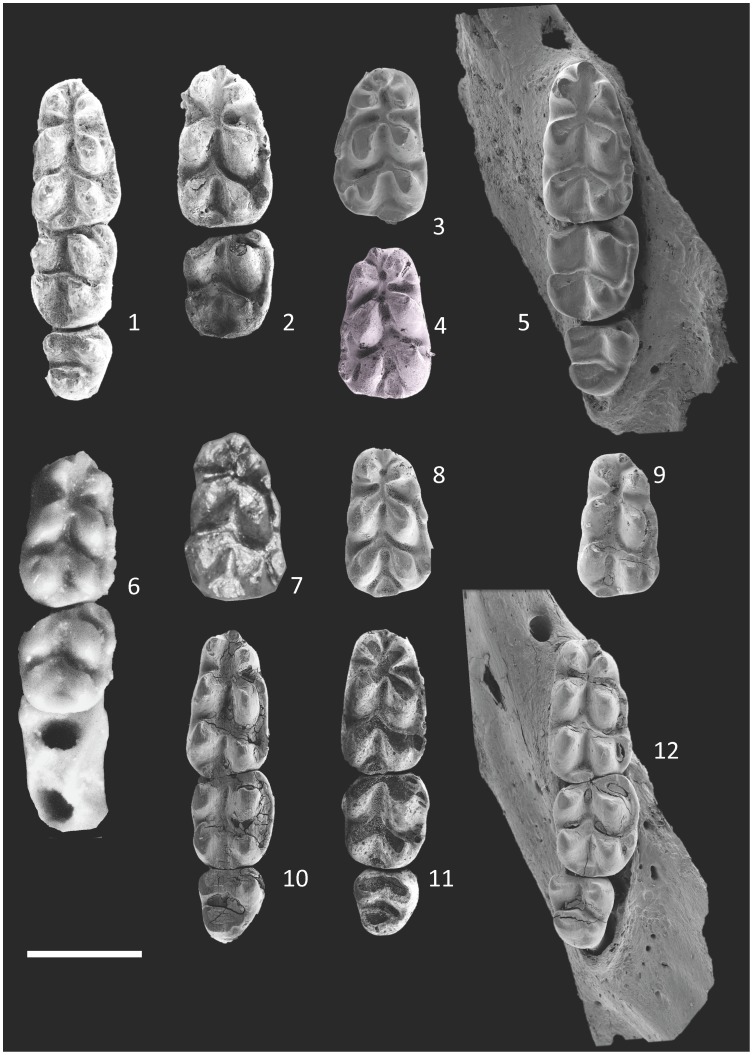
Mandibular molars of *Micromys praeminutus* (1–8) and *Micromys minutus* (9–12): 1– m1-m3 MN17 Včeláre 6/1/17, 2– m1-m2 MN17 Včeláere 6/1/16, 3– m1 MN17 Včeláre 6/1/25, 4– m1 MN17 Včeláre 6/1/27, 5– m1-3 MN17 Včeláre 6/1/18, 6– m1-m2 Zhevachova Gora 15, 7– m1 Gundersheim-Findling, 8– m1 MN7 Včeláre 6/1/26, 9 - Q4 Zazděná 2/2, 10–Recent CZ, ISZ M50, 11–Q4 Zazděná 2/4, 12– Recent CZ ISZ M121. Scale 1 mm.

**Figure 8:**
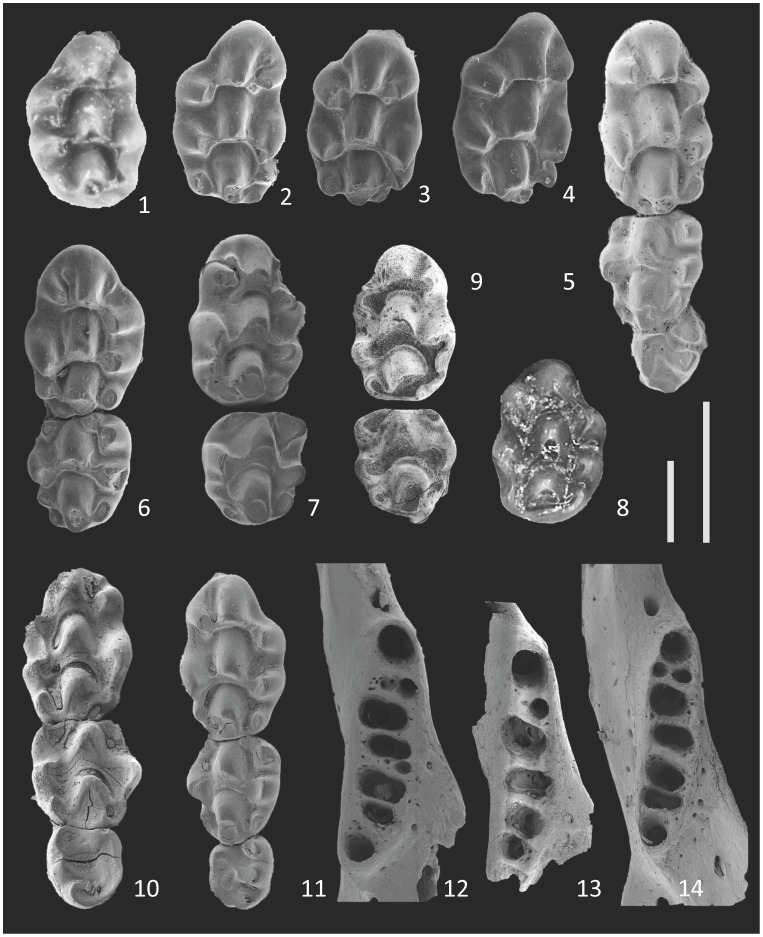
Maxillary molars of *Micromys praeminutus* (1–8) and *Micromys minutus* (9–11) and toothless mandibles of *Micromys praeminutus* (12) and *Micromys minutus* (13–14): 1- M1 Csarnóta 2, type, 2– M1 Včeláre 6/1/11, 3 - M1 Včeláre 6/1/12, 4 - M1 Včeláre 6/1/1, 5 - M1-M3 Včeláre 6/1/4, 6 - M1-M2 Včeláre 6/1/8, 7 - M1 Včeláre 6/1/7, 8– M1 Hohensülzen, 9– M1-M2 Zazděná 2/3, 10– M1–M3 Recent CZ ISZ M50, 11– M1–M3 Recent CZ ISZ M108, 12– md s. (inv.) Včeláre 6/1/21, 13– md d. Zazděná 2/1, 14– md d. Recent CZ ISZ M108. Scale 1 mm - right for 1–11, left for 12–14.

**Figure 9:**
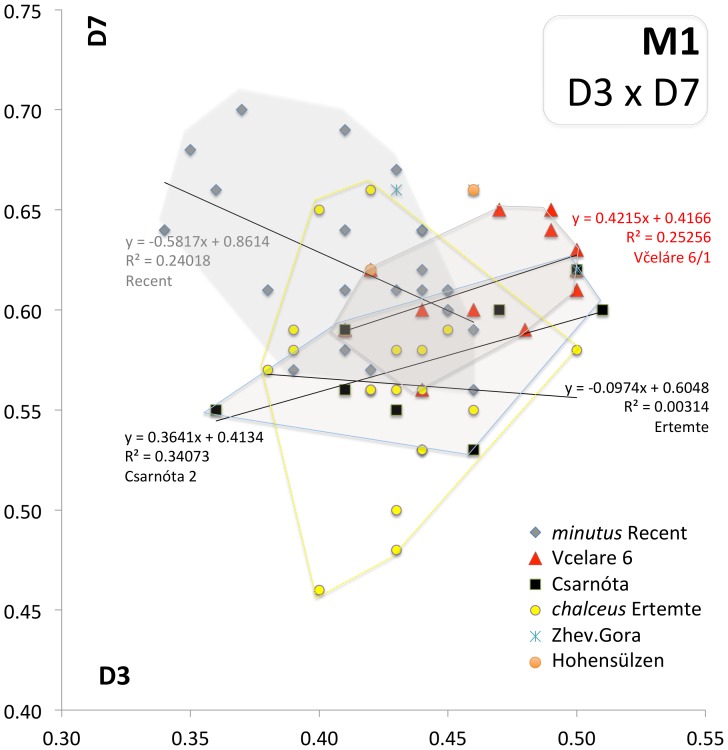
Scatter plot of D3 and D7 dimensions (characterizing a shape of central cusp of M1– t5) and corresponding regression lines in selected populations of *Micromys.*

Morphometrical data obtained from particular samples are (for selected variables) summarized in Tables S1A–B and Figs. 5, 6, 7, 8, 9, 10, 11. As found in previous comparisons, particular populations and presumed species differ in tooth size both in terms of their average and extreme values (Fig 5, 6). All studied samples exhibit a derived state of major generic characters (5-rooted M1 with distal position of t1, cusp-like t7, absence of distinct t1bis or t4bis, i.e. additional cusps at t1 or t4 ridges, 3/4/−rooted m1 with tma and cingular ridge – Fig. 7,8), but a relatively broad span of metric variation. Yet, there is a considerable overlap in the state of both metric and non-metric variables among particular populations (including the extant *minutus* and fossil forms). To analyze the meaning of differences between individual samples we first had to examine the variation in particular dental characters and assess their contribution to between-population and between-strata variations.

### (a) Variation Pattern of Dental Phenotype

#### M1

The longitudinal metrical variables are significantly correlated (r>0.5, p<0.001) with total length of the tooth (D1). The same is valid for the largest width of the tooth (D4, D5), though not for other latitudinal tooth variables. The differences in the former variables were found to be the most prominent source of the between-population and between-strata total variance (ANOVA). Yet, there is a considerable overlap in the variation ranges of particular populations in all metrical variables. This concerns also the M1 latitudinal variables D6–D11, whose contribution to between-population variation was found insignificant in general, though their state and pattern of variation might be quite specific in particular populations. For instance, there is no significant correlation between the length of the t5 complex (D3) and t4–t6 width (D7) in the total sample (r = 0.087), though these variables show significant correlations within particular population samples. Moreover, the shape of the respective relation (as expressed by slope of regression) discriminates between particular populations quite markedly (Fig. 9). It is worth mentioning here that the samples from MN17 Včeláre 6 and MN15 Csarnóta are almost identical in this respect, while the Recent sample as well as MN13 *M.chalceus* differ from them quite distinctly.

The following non-metrical variables were found to produce the most significant effects (all p<0.00001) upon the between-sample variation: *t7pos* (R = 0.905, F = 23.481), *t7size* (R = 0.810, F = 9.880), *t4/7sep* (R = 0.789, F = 5.524), *t9size* (R = 0.760, F = 6.898), *t1–2shap* (R = 0.760, F = 7.006), *t6/9size* (R = 0.719, F = 6.105), and *t6/9sep* (R = 0.709, F = 5.212). In contrast, no significant effects were found in *t6size*, *t0, t3spur,* and *t12pos*. In short, particular fossil populations and the Recent sample differ mainly in the size and position of t7 and t9.

Correspondingly, in the analysis of principal components (Fig. 10), t9 related variables were by far the most essential component of the factor variable 1 (with factor loadings of 0.923 for *t9size*, 0.914 for *t6/9size*, and 0.693 for *t6/9sep*), while for the factor 2 the same was true of t7 variables (*t7pos t7size* 0.867, *t4/7sep* 0.760) and *t1–2shap* (0.752).

On **m1**, the main dimensions both longitudinal and latitudinal, i.e. d1, d4 and d5, d6 were mutually correlated (r>0.7, p<0.001), while those characterizing the length of the anteroconid complex (d2, d3) were only weakly correlated (r<0.4, p>0.05) either to the main dimensions or to each other. Yet, d3– the posterior length of the anteroconid complex – was together with d1 and d4 by far the most significant source of the between-sample and between-strata variation (ANOVA: d1 R = 0.710, F = 15.840, d4 R = 0.627, F = 10.101, d3 R = 0.602, F = 9.623). Among the non-metric variables, only *cingSIZE* and *cingCUSP,* and *tmaPOS* and *ASYM* were significantly correlated (r = 0.626 and r = 0.372, p<0.001), while the correlation with metric variables was only weak. Significant values (p<0.001) appeared only in the following cases: *tmaSIZE* with d3 (r = 0.433), *cingCUSP* (not *cingSIZE*) with d5 (r = 0.378) and at a level of r<0.3 also between d6,d10,d11, *tmaPOS* and d1 (r = −0.267). The following variables were found to contribute significantly to between-sample and between-strata variation: *cingCUSP* (R = 0.539, F = 6.919), *cingSIZE* (R = 0.527, F = 5.992), and *tmaSIZE* (R = 0.514, F = 5.008).

In contrast, no significant effect (R<0.2, p>0.05) upon the between-sample and between-strata variation was found in the following variables: d2, d7, d9, d13-15, *ASYM, tmaPOS*, i.e. the variables characterizing the arrangement of the anterior part of the anteroconid complex (besides the size of tma) and the length of the talonidal complex.

Consequently, apart from the general size and proportion of the tooth, the total length of the anteroconid complex, the size of tma, and the size and shape of the cingular ridge seem to be the characters of the highest presumptive taxonomic value.

### (b) Between-sample Comparisons

In regard to the state of the characters significantly contributing to the between-sample variation (see above), each population can be characterized as follows:

The model population from the MN17 site Včeláre 6/1 is characterized by the following:

- well pronounced tma in all m1; in some, the tma is even quite large (comp. Fig. 8– Vcel6/1/18);
- a broad cingular shelf with well-marked c1, in three individuals all cusps c1–c4;
- large, cusp-like t7, mostly in a medium position parallel to t8, the ridge connecting t7 and t8 weak only;
- t9 smaller than t6 but separated with a deep valley in most specimens.

There is a broad measure of correspondence between the population of Včeláre 6/1 and samples from Zhevachova Gora, Vinogradovka 2, and Beremend 11, both in the mean state of the above listed characters and in their variation pattern. Also, a single M1 from Gundersheim (Heller’s collection) falls within the variation span of the Včeláre 6 population.

With certain differences, this also holds for the type series of *M. praeminutus* from MN15 Csarnóta 2:

- in all but two specimens tma is present, though smaller on average than in Včeláre 6, and situated rather in a central mesial position;
- compared to MN17 samples, t7 is somewhat less pronounced and situated in a more mesial position;
- t9 is large, in a third of specimens it is of equal size to t6, though not completely separated.

Specimens from Kotlovina 3 and Vinogradovka 3 exhibit just these specific differences. Nevertheless, there is obviously no categorical difference between the type series of *praeminutus* and the MN17 samples. Both metrical and non-metrical characters show considerable overlap and no significant differences in mean values were found.

More pronounced differences were found in four remarkably robust teeth (2 M1, 2 m1) from Gundersheim-Findling (comp. [22], Abb. 51–53) and also in some specimens from Ręmbielice Królewskie: two of 4 m1 from Ręmbielice I are hypsodont with clearly separated major cusps and a cingular ridge split into distinct and large cingular cuspids resembling the phenotype of *M. cingulatus*. One M1 corresponds to *M. praeminutus* except for a rather small t9 and indistinct t7; the other M1s originally identified as “*Micromys* ?” are large with nearly complete stephanodonty (confluence of distal cusps) and supposedly do not belong to that genus.

The extant form, *M. minutus*, exhibits, in regard to overall character variation in the set of studied samples, the following specificities: (1) t9 is quite reduced and only weakly separated from t6; even on unworn teeth both cusps are connected with a distinct ridge; (2) t7 is situated in a distal position forming the distopalatal margin of the tooth crown (both on M1 and M2); the size of t7 varies – in one tooth, t7 is completely absent; (3) t12 is minute as a rule, not of a cusp-like appearance, but contributing to the ridge which connects t7 and t8 and forming the perpendicular distal margin of the tooth; (4) t12 is absent on M2; (5) t3 on M2 is reduced or indistinct; (6) the cingular ridge on m1 is clearly distinguished but narrow, while individual cingular cusps are quite indistinct; (7) the same is true of the cingular ridge on m2, where it includes the labial anteroconid, which is much smaller than the protoconid; (8) tma is always present, not displaced from a central anterior position though small as a rule.

In contrast to the Weichselian and/or Holocene fossils (Tarkö, Rejtek, Soutěska, Zazděná, Bašta), which fit well to the variation span of the Recent population (Fig. 7,8), the Biharian M1s from Hohensülzen differ by their relatively large t9, distinctly separated from t6, and the absence of t12, while the m1s from the Zamkowa Dolna Cave differ in their robust cingular ridge which is distinctly broader at well-marked c1. In these regards, the specimens from the above-mentioned Biharian sites correspond more to the MN15–MN17 species.

Multivariate analysis (PCA) demonstrated a broad overlap among particular Pliocene and Early Pleistocene populations and a distinct position of Recent *M. minutus* and Chinese Turolian *M. chalceus* (Fig. 11). With respect to range of variation, the former samples correspond roughly to those of the latter species. These results are in good agreement with the broad overlap and general correspondence among the examined Pliocene and Early Pleistocene populations with respect to the state of the critical taxonomically significant characters (t7, t9, tma, cingular ridge, number of roots on M1 and m1).

Consequently, with an acceptable level of reliability, it appears that the studied material is composed of only three distinct entities, viz. MN13 *chalceus*, Recent *minutus* (including the Late Pleistocene and Holocene items), and the MN15-Q2 form, which includes the type series of *praeminutus* Kretzoi, 1959, this providing an available default name for it.

## Discussion

The genus *Micromys* first appeared in the late Miocene in China (*M. chalceus* Storch, 1987: Ertemte). In Europe, the genus is reported to have appeared at nearly the same time, i.e. along the Turolian-Ruscinian (MN13/MN14) boundary (comp. *M*. *paricioi* Mein, Moissenet and Adrover, 1983: Celadas 4, *M. cingulatus* Storch and Dahlmann, 1995: Maramena), although recent records suggest that it might have appeared there already during the earlier stage of MN13 (comp. *M*. *paricioi* in Granada Basin: [37]). In any case, during the Ruscinian it exhibited considerable diversification, especially in the Mediterranean region [5]–[6], [8]–[9]. The appearance of the large-sized forms, each well distinguished also in terms of categorical characters, namely *M. steffensi* van de Weerd, 1979, *M. bendai* van de Weerd, 1979, and *M. cingulatus* Storch and Dahlmann, 1995 illustrates this fact quite convincingly. Yet, the vast majority of the representatives of the genus reported from the European Ruscinian and Vilanyian sites (see Dataset S1) fall into the category of smaller or medium-sized forms whose taxonomic status is often considered rather confused. In most instances they have been co-identified with *M. praeminutus* Kretzoi, 1959 (described from MN15 site Csarnóta 2 in S-Hungary), the form traditionally believed to represent a transitional stage between the Early Pliocene radiations and the extant species ([12]–[13], [17] and others). Yet some authors (comp. e.g. a review by Kowalski 2001) consider the validity of the species status of *praeminutus* doubtful and co-identify the respective Pliocene and Quaternary items just with the extant species.

The present paper is intended to explain the discrepancies and refine information on this topic by means of a detailed reexamination of a significant part of the Pliocene and Quaternary record of the genus from Central Europe. Yet, except for the few cases discussed below, we found a considerable overlap among particular fossil populations in both metrical and non-metrical characters, but quite distinct differences between them and the Recent species, as well as the Chinese Turolian *M. chalceus*. To discuss the meaning of these results we have first to confront them with the diagnostic setting of the genus and aspects of its phylogenetic morphocline.

The odontological characteristics of the genus *Micromys* supplementing the diagnosis provided by Storch [29] would include the following specificities: (i) very small size and brachyodont molars; (ii) relatively narrow occlusal outline of M1; (iii) mesial cusps t1–3 are distinctly separated from the distal complex; t1 and t4 as well as t3 and t6 are separated by broad valleys; no interconnecting ridges are present; (iv) t1 is in a distal position as a rule, t3 is closely crowded against t2; (v) on m1 the anteroconid cusps and medial cusps (met-pro) are connected with a narrow medial ridge, the lingual anteroconid is slightly larger compared to the labial one and displaced anteriorly; (vi) tma is present, as a rule; (vii) there is a distinct cingular ridge on m1 and m2, which integrates the relatively less differentiated cingular cusps c1–c4 and forms a nearly continuous structure along the buccal margin of teeth crowns; (viii) t7 is well developed on M1and M2, and distinctly separated both from t8 and t4; (ix) m1 with minute additional lateral root(s) between the main mesial and distal root; (x) M1 with 5 roots. As demonstrated elsewhere ( [29], [9]) the characters (viii)–(x) may be absent in the Turolian and early Ruscinian populations of the genus. The earliest taxa of the genus (*M. paricioi* and *M. chalceus*) differ from other members of the genus by a weak cingular ridge on m1 and m2, a minute t7 or its complete absence, a relatively large t9 deeply separated from t6, and 3 roots on M1.

The morphocline characterizing the Pliocene history of the dental phylogeny of the genus includes (a) the reduction of t9 on M1, (b) the development of t7 on M1–M2, (c) an increase in root number on M1, (d) the narrowing of M1 and m1 with (e) the fusion of cingular cusps on m1 and m2 onto a continuous cingular ridge. Most of these transformations took place around the Miocene/Pliocene boundary, MN13–MN15. Whether they continued in the same directions and at the same rates also during the Pliocene and Early Pleistocene (terminating in the phenotype characterizing the extant species), or whether the fossil record suggests rather a discontinuity between the phenotypic variation of the extant species and fossil populations is not entirely clear. To answer these questions was a primary motivation of the present study.

In general, except for few misidentified items, the material under study exhibited complete agreement with the above-mentioned diagnostic characters of the genus. In comparison to the Early Ruscinian or Turolian forms, it shows derived states of the variable characters (viii–x) and (b)–(e). Comparisons of the Early Pleistocene, MN17 and MN16-15 samples suggested possible trends in the percentage of plesiomorphic morphotypes (such as the mesial position of t7), but, in general, it seems that the within-population variation in the state of particular characters exceeds the level of mean differences between particular populations. In other words, in the frame of the examined Pliocene and Early Pleistocene sample, we did not find a clear time-dependent morphocline in any character under study, perhaps except for the somewhat smaller size of the Pleistocene items. Unfortunately, the Quaternary record of the genus is limited to a few isolated teeth and real between-population comparison is not available. The well pronounced differences between the set of Pliocene and Early Pleistocene fossils and the Recent form (e.g. in the massive reduction of t9, the distal position of t7, the reduction in tma, and the smaller size of the latter) robustly supports a clear taxonomic distinction between them but in no way the hypothesis concerning subsequent gradual rearrangements and/or the appearance of continuous phenotype morphoclines.

### (i) Phylogenetic Morphocline of the Genus, Status of *M. praeminutus*

The most recent and most detailed discussion on the phenotypic specificities of the Pliocene *Micromys* was undertaken by Minwer-Barakat et al. [10], based on an extensive study of a large amount of material from the MN16 site Tollo de Chiclana 13, Gaudix Basin, Spain. The authors demonstrated convincingly the differences of the fossil population from the extant species (stressing the above-mentioned characters, particularly the well-developed t9 on M1 and the distinct t12 on M1 and M2 in the fossil form), as well as its differences from all (until then) named fossil species. Consequently, the respective population was described as a new species, *M. caesaris* Minwer-Barakat et al., 2008. By direct comparison, Minwer-Barakat et al. [10] demonstrated that the material from the Late Pliocene sites Valdeganga 9b,7 [17], Mas Rambault 2 [38], and the MN17 Tegelen formation in Zuurland boreholes [14] formerly identified as *M. praeminutus* are almost identical with the newly described species and anticipated the same also for other localities. Indeed, our material from Včeláre 6 (similarly to other MN17 samples in our material) fits the diagnostic features of *M. caesaris* perfectly. Yet, we also found a broad measure of correspondence between our series and the type series of *M. praeminutus* from Csarnóta, and tend to believe that the same is the case with the relations between type populations of *praeminutus* sensu stricto and *caesaris*. Obviously, this holds true for metrical characters but, as pointed out by Minwer-Barakat et al. [10], “morphologically, *M. caesaris* can be distinguished from *M. praeminutus* in several characters, especially the presence of a large tma in m1; on the other hand, *M. caesaris* differs from *M. minutus* in the development of t9 and t12 in M1 and M2. ” Here, we demonstrated that the characters on which the diagnosis of *M. caesaris* was based exhibit a broad variation within particular MN15–MN17 populations, and the states predicted for *M. praeminutus* do not in fact characterize the type series, while the mean state of the respective variables is nearly the same as reported for *caesaris*.

Obviously, the description of *caesaris* has been influenced by the confused interpretation of *M. praeminutus*. The confusion arose primarily because of the original description by M. Kretzoi [11], which was as follows: “*Micromys praeminutus* n. sp. – Einige Molaren einer sehr kleinen Muridenform erinnern in Kauflächenbild an *Apodemus*, doch lassen sie sich von dieser Gattung durch sehr kleine Dimensionen und praktisches Fehlen der accesorischen Höckerchen (an ihrer Stelle is bloss eine schwache Leiste wahrzunehmen) gut unterscheiden und liefern gleichzeitig einen Beweis für die Richtigkeit einer Einordnung dieser Form in die – abgesehen von einer nordchinesischen biharischen Angabe (Choukoutien) – erst seit dem angehende Holozän bekannte Gattung *Micromys* liefern.” [“*Micromys praeminutus* n. sp. – Several molars of a very small murid resembling *Apodemus* in occlusal pattern yet differing by much smaller dimensions and absence of cingular cusps (instead of which they bear a continuous crest). These characters clearly distinguish the form which represents the first fossil record of *Micromys*, the genus known from the Holocene only (except for a single Biharian records from N Chinese site Choukoutien)].

The respective diagnosis stressed the generic assignment of the respective fossil to *Micromys* and its difference from the extant species. Although in both respects it characterized the respective form quite realistically (for this reason the name is undoubtedly not a *nomen nudum*), obviously it was too brief to exclude further misunderstanding. Sulimski [39] reporting the species from the MN15 site Węże 1 did not extend the diagnoses in essence. Thus, the first detailed description was perhaps that by Michaux [12], who reported 2 M1, 2 m1 and 1 m2 from the MN15 site Sète, in which he pointed out the well-developed t7 and t9, the weak development of the cingular ridge (compared to *Apodemus*), and in particular the absence of tma. Correspondingly, the absence of tma has also been considered a typical character of *M. praeminutus* by further authors [17], [40] and has become a major feature also in the differential diagnosis of *M. caesaris*. Of course, it cannot be excluded that the absence of tma actually appeared in some populations (e.g. Sète) and some of them represented separate species. Yet, obviously this was not the case with type series of *praeminutus,* the sample from MN15 Węże 1(comp. [39]) or other populations under our study.

In short, accepting a broader phenotypic delimitation of particular fossil taxa as suggested in this paper, *M. caesaris* does not seem to differ essentially from the type population of *M. praeminutus*; thus, *caesaris* Minwer-Barakat et al., 2008 should be considered a younger synonym of *praeminutus* Kretzoi, 1959.

In addition, it is worth mentioning that Schaub [41], who did not take into account the possibility of a Pliocene appearance of *Micromys* in Europe, attributed to his genus *Parapodemus* Schaub, 1938, a species described as *P. coronensis* Schaub, 1938 from the Middle Pleistocene ?/Biharian site Brasso (based on a single mandible). Storch and Dahlman [9] argue that the type specimen of *coronensis* should clearly be assigned to *Micromys* (despite the fact that it is heavily worn and some features are hard to judge in detail). Accepting this proposal (which seems to be well substantiated), in regard to the results of the present study suggesting the taxonomic homogeneity of the Late Pliocene-Middle Pleistocene *Micromys* in Central Europe, *coronensis* Schaub, 1938 should be considered a valid name of the taxon (with *M. praeminutus* Kretzoi, 1959 and *M. caesaris* Minwer-Barakat et al., 2008 as its junior synonyma). Yet, without a direct revision of the respective types, such a solution should be considered merely as a working hypothesis.

### (ii) Fossil Record and Molecular Data Come into Accord

In conclusion, our study demonstrated the following: (1) Considerable differences exist between the European fossil forms and the extant representative of the genus (monotypic species *M. minutus)*. (2) The vast majority of the Pliocene and Early Pleistocene European populations can be considered as a single entity, including the type series of *M. praeminutus*. (3) The Early Pliocene record of the genus is relatively rich and suggests a clear phylogenetic radiation (in our material comp. distinct forms in Gundersheim-Findling and Ręmbielice Królewskie II). (4) The rarity of the genus in the Early Pleistocene fossil record suggests its actual disappearance during that time. (5) In contrast to the Middle Pleistocene sites where the genus is almost missing, in the Vistulian and Holocene, it appears in several localities in Central Europe. The respective individuals clearly correspond to the Recent species.

We could finish our report at this point. Yet, the most exciting issues of this topic would remain untouched. These concern the discrepancies between the fossil record (or, to be exact, its default interpretation) and the view of the genus through the optics of the molecular phylogeny and phylogeography of the genus. Our results suggest that at least some of them need not be too great, in fact. Conclusion (5), indicating expansion of the recent species during the Late Pleistocene-Holocene, seems to be in very good agreement with molecular dating (80 ky) inferred of the surprising genetic homogeneity of *Micromys minutus* throughout the whole its contemporary range, i.e. from Britain to Japan [4]. In contrast to traditional expectations concerning the continuous appearance of the genus in Europe and the gradual transformation of the Pliocene taxon into the extant one, our results suggest that the fossil record does not provide reliable support for such a hypothesis. Rather, it indicates that the European Pliocene form *praeminutus/coronensis* and the Late Pleistocene–Recent *minutus* are distinct phylogenetic entities, and that the Early Pleistocene records indicating relic appearance of the genus in Europe more likely belong to the former taxon than to the extant species.

As an attempt to explain the discrepancy between the traditional view and the output of molecular studies, Yasuda et al. [4] propose a hypothesis on repeated range expansion-contraction events responding to the glacial/interglacial cycles of the Quaternary past. Of course, no direct support of this hypothesis is available (either from fossil records or molecular data); also its indirect support is rather weak. In contrast to the Late Pleistocene, which clearly involved a large scale range dynamic in response to climatic oscillations that undoubtedly concerned almost all faunal elements, Early Pleistocene faunal history is characterized by nearly constant taxonomic composition, high species diversity, and more or less stable community structure during the course of a glacial cycle. Thus, in the Early Pleistocene, expansion/extinction events were obviously rather rare and cannot be taken as a default model of Early Pleistocene range dynamics [20]. In contrast to the Late Pleistocene-Holocene, the European Early Pleistocene records of *Micromys* are extremely rare and show no clear pattern of aggregation in particular time horizons (which could indicate expansion events). Thus, for the Early Pleistocene history of the genus, the fossil record more likely suggests a vicariance dynamic with islet-like persistence of resident refugial populations throughout Europe rather than periodical extinctions and recolonizations over a major part of its eupalearctic range.

At least for these reasons it seems more reasonable to expect that the European Pliocene and Early Pleistocene populations of the genus *Micromys* represented descendant(s) of local clades resident in the Western Palearctic from the Late Miocene. In any case, it seems evident that the patterns of the Late Miocene – Pliocene phenotypic evolution of the genus in Europe differ from those appearing in the Chinese fossil record represented by forms like *M. chalceus*, *tedfordi* and *minutus* (comp. e.g. a minute tma, reduced t12 and cingular cusps), which might indicate independent parallel evolution in the Western and Eastern part of the range. Tentatively, we can hypothesize quite a deep divergence of the two clades, supposedly even during the first spread of the genus in the Late Miocene, followed by abrupt radiation in Europe during the Ruscinian. If so, the respective phylogenetic setting could be worthy of a taxonomic expression, i.e. the European clade should be classified in a separate genus.

The molecular phylogenetic analyses repeatedly demonstrated that the genus *Micromys* was produced by a basal divergence of Rattini, i.e. one of the deepest branches of the family radiation [1]–[3]. The default hypothesis on its origin from early stock of *Apodemus* radiation during MN13 (comp. e.g. [6]) seems to be thus quite improbable. Molecular dating places the divergence of *Rattus/Mus* (i.e. Rattini/non-Rattini murids) in the interval 8.3–10.6 Ma [1]. As *Micromys* represents the deepest branch within Rattini we can hypothesize that the ancestor clade of *Micromys* was established soon after the initial divergence, i.e. during MN10–MN12. The phenotypic characteristics of the expected ancestor can be tentatively estimated from the situation in the earliest representatives of the genus, i.e. MN 13 *M. chalceus* in China and *M. paricioi* in Spain, or *M. cingulatus* in the Eastern Mediterranean. The former two were small forms exhibiting brachyodont molars with incomplete development of t7 (this often situated at a mesial position close to t4), large t9 (at a mesial position), a weak cingular ridge on m1 and m2, and incipient tma. The latter differs by having more robust teeth and prominent cingular cusps, tma and t12. Correspondingly, the Late Turolian-Early Ruscinian (MN13/MN14) forms from the Mediterranean region (*M. cingulatus, M. steffensi, M. bendai*) show considerable phenotypic divergence, all with well-developed t7, a marked cingular ridge and the appearance of additional roots on M1 and m1.

Of course, it should be remembered that the trends characterizing the early history of *Micromys* in Europe can also be observed in the early stages of dental evolution in other clades of murids (e.g. *Apodemus, Rhagapodemus* or *Stephanomys*, particularly when compared to their expected ancestor such as *Progonomys*). A level of differentiation of the respective characters is often considered as a general marker of the progressivity level of a particular murid clade [42]–[44]. In short, the possibility that the same morphoclines and nearly identical phenotypes might appear in quite distant clades of early murid radiation cannot be excluded and in the following considerations it will be taken in account. In any case, just for these reasons, a search for ancestral forms of particular clades is obviously an extremely difficult task. Yet, in regard to the above-mentioned phenotypic setting of the earliest *Micromys*, its hypothetical ancestor can be sought among the small-sized generalized forms with incipient stages of the derived characters of early *Micromys* s.str. Essentially, there are two genera in the Western Palearctics which should be taken into account here: *Progonomys* and *Parapodemus.* The forms attributed to them constituted the dominant component of the Valesian and Turolian murid assemblages.

The genus *Progonomys* was described by Schaub (1938, pp.19–21) [41] (with a type species *Progonomys cathalai* Schaub, 1938 - type locality: MN10 Montredon) as an ancestral grade of the family, differing from true cricetids by a typical murid arrangement of cusps (e.g. X-pattern of the anteroconid complex) but the absence of derived dental characters of modern clades (such as tma, t7, or a connection between t6 and t9). A vast majority of further records of the family from the European Late Miocene was co-identified only with this genus. The forms arranged in it are characterized by the absence of t7 and a ridge between t4 and t8; a small t12 at a distal position; well-developed and cusp-like t9 distinctly separated from t6; a variable position of t1 and shape of the t1–2 margin of the tooth; the general absence of t1bis and t4bis or their appearance in a small proportion of populations; broad m1, mostly without tma; nearly symmetrical anteroconids; and distinct c1 and c2 together with entostylids at the labial side of the tooth (comp. [45]–[47] and others).

An extensive revision of the genus was undertaken by Mein et al. [45], who stressed that “*Progonomys,* in its previous concept, was a clearly paraphyletic genus that included numerous species, brought together on the basis of plesiomorphic characters.” After the exclusion of *Progonomys hispanicus* Michaux, 1971 (placed in the genus *Occitanomys* as its ancestral form); *P. clauzoni* Aguilar et al, 1986; *P. debruijni* Jacobs, 1978 from Pakistan; and Chinese *P. yunannensis* Qui and Storch, 1990, they suggested that the genus *Progonomys* includes just three species: *Progonomys* sp. from MN9 Sinap, Turkey; *P. cathalai* Schaub, 1938 from a number of Anatolian and W-European MN10 sites; and *P. woelferi* Bachmayer & Wilson, 1970 from Kohfidish and other late MN10 sites, all representing a continuous ancestor-descendant lineage. Mein et al. [45] further argued that the trend of size increase characterizing the morphocline of the genus during MN9–MN11 (from *P. cathalai* to *P. woelferi*) was further accompanied by a divergence of large-sized forms with interconnected m1 anteroconids, an anterior position of t1, and particular cusps of large size, which they place in a separate genus *Huerzelerimys* (type species *Parapodemus vireti* Schaub, 1938 from MN 11 Mollon (Ain), France).

Yet the proposal by Mein et al. [45] formally simplifying the situation was not generally accepted. Similarly to [48], van Dam [43], who analyzed the relations of *Progonomys hispanicus* and *Occitnomys brailloni* in detail, argued that the former taxon is much closer to *P. cathalai* than to the latter and proposed its inclusion in the genus *Progonomys*, though he stressed its essential difference from *P. cathalai* in the position of t9 (distal in *P. hispanicus-Occitanomys* clade). Storch and Ni [49] demonstrated that the corresponding trends in the Chinese fossil record appeared parallel to those in the West Palearctic populations and illustrated this with the case of *Progonomys yunnanensis*, which they placed into a new genus *Linomys*. Sen [50] contributed further data on the early Vallesian (MN9) record from Sinap Tepe, Turkey, covering *P. cathalai* and a small-sized form newly described as *P. minus* Sen, 2003. As a separate genus, he distinguished a large-size form with a confluent pattern of major cusps in M1 (*Sinapodemus ibrahimi*). An extensive revision and survey of the genus *Progonomys* was recently provided by Wessels ([47], pp. 89–125), who stressed the extremely broad phenotypic variation in most populations and finally arranged the vast majority of them under the type species, *P. cathalai*. Along with it, she included three other valid species in the genus: *P. woelferi* (as the most derived), *P. hispanicus*, and *P. debruijni* (including *P. minus*). Among others, she synonymized *Sinapodemus ibrahimi* Sen, 2003 and *Progonomys sinensis* Qiu, Zheng et Zhang, 2004 with *P. cathalai* and proposed that *Progonomys clauzoni* Aguilar, Calvet et Michaux, 1986 exceeds the variation in these species and should be removed from the genus.

A detailed analysis of the variation pattern in large populations of *Progonomys clauzoni* undertaken by Lazzari et al. [36] demonstrated an extraordinarily broad span of independent variation in almost all indexing dental characters, including the diagnostic characters of early *Micromys* or *Parapodemus,* and suggested that unconstrained variation in these characters is quite typical for the early stage of murid radiation.

In the light of all this, it seems that it is beyond the scope of morphomeric comparison to find the true roots of extant murid clades, and the well substantiated attempts by Mein [31], [45] to resolve the confusing view of the early radiation of murids by identifying clearly defined monophyla need not always produce entirely satisfying results.

Of course, in the search for the ancestry of European Mio-Pliocene *Micromys,* another group of small-sized murids accompanying *Progonomys* at many sites must also be taken into account. Namely, it is the genus *Parapodemus*, composed of small-sized forms with t6 and t9 weakly separated and of similar size, well developed tma in a central anterior position forming a “trifolium” pattern in worn teeth [39], and t4 connected to t8 by a high and continuous crest (in *P. lugdunensis)* or with t7 at that position (i.e. at mesio-palatal base of t8), as it is in the type species of the genus, *P. gaudryi* (Dames, 1883) (MN 12 Pikermi) and the younger forms referred to the genus. Such a position for t7 is typical also for the early forms of *Micromys* (*cingulatus, steffensi, bendai*). The cingular ridge is well pronounced in *Parapodemus* but, as a rule, c1 is distinct and larger than tma. Martín-Suárez and Freudenthal [42] report that in the earliest form of the genus MN 11 *P. lugdunensis* a small medium root in m1 (conforming to the state in *Micromys*) is present in 60% of individuals. In general, the Late Miocene populations of the genus (mostly referred to *P. lugdunensis*) are characterized by a broad variation range, typically centered with the distal position of t1, large t9 and t12, and a ridge-like incipient t7.

The genus *Parapodemus* has been something of a puzzle in the taxonomy of fossil murids. Schaub [41] described the genus based on *Mus (Acomys?) gaudryi* Dames, 1883 from MN 12 Pikermi and suggested it as a direct ancestor of *Apodemus*, from which it differed by its smaller size and the more slender construction of its molars (including more pronounced brachyodonty, the less marked emancipation of particular cusps including t7, and a lesser degree of stephanodonty). He included in the species also a mandible from MN 13 Polgardi reported by Kormos [41], which was later separated by Papp [52] into an independent species *Parapodemus schaubi* Papp, 1947, and another newly described form, *P. lugdunensis* from MN11 Mollon, France. The small-sized brachyodont murids with undeveloped t7 co-identified with *P. lugdunensis* represent together with *Progonomys* spp. the dominant components of the European murid communities of the Late Miocene (Kohfidish, Dorn-Dürkheim, Eichkögel, jask. Mala a.o.) including the earliest murids from MN9 sites in Moldovia and Ukraina [53], [23].

The inconsistent setting of the taxon and confusing diagnosis led Mein [54] to a proposal to synonymize *Parapodemus* with *Apodemus*, a view which was immediately criticized by van de Weerd and de Bruijn [55]. Yet, finally, Martín-Suárez and Mein [56] argued on the generic identity of derived forms of *Parapodemus* and *Apodemus* and proposed to include all named taxa of *Parapodemus* except for the type species of the genus into *Apodemus* as a stem line of the respective panmonophylum. Such a solution, essentially simplifying the confused interpretation of the whole group, has been widely accepted (comp. [44]). As stressed by Martín-Suárez and Mein [56], the early history of the clade is characterized by a gradual increase in size, hypsodonty, and a degree of transversal integration of main cusps in the populations attributed to *Parapodemus* s.l. during the period MN10–MN12 ([42]). In respect to this, the succession of *P. lugdunensis* Schaub,1938– *P. barbarae* van de Weerd, 1976– *P. meini* Martín-Suárez et Freudenthal, 1993– *Apodemus gudrunae* van de Weerd, 1976 has been identified as a continuous ancestor-descendant lineage of prominent use in Late Miocene mammal biostratigraphy [57], [34]. Its terminal elements in MN13 exhibit a broad measure of agreement with the diagnostic criteria of the genus *Apodemus* (viz., t1 in a proximal position; t6 and t9 of equal size and in direct contact; the presence of t7 and/or a ridge connecting t4 and t8; t7 smaller than t9 and often at a proximal position when of a cusp-like form (i.e. at the mesio-labial base of t8); t12 cusp-like at the disto-labial position; m1 with tma; anteroconid complex symmetric; robust cingular cusps, mutually separated but not interconnected with a common ridge).

Yet, at least some of the small-sized brachyodont forms placed in the genus *Parapodemus* exhibit a large number of the characters expected in the ancestors of the West Palearctic *Micromys* (see above), and one cannot exclude that the respective population included the elements actually belonging to that stock. It is worth mentioning that such a possibility arises with the type species of the genus, *Parapodemus gaudryi* (Dames, 1883), including its neotype figured in [43] - comp. incipient t7, t3 spur, weak c1 but distinct cingular ridge etc. [58], [43]. Correspondingly, the appearance of additional roots in m1, or the shape of anteroconids and tma on m1 reported in MN14-15 populations identified as *Parapodemus schaubi* (e.g. in Węże or Podlesice – comp. [39]) conform quite well to the trends characterizing the Late Miocene-Early Pliocene stage of *Micromys* radiation (see above).

Consequently, if the earliest stages of Rattini radiation appeared also in the Western Palearctic (which seems quite probable), one can expect here both (a) the clade establishing the *Rattus*-like dental rearrangements, e.g. perpendicular cusp confluences on M1 (comp. *Sinapodemus* Sen, 2003), and (b) the small sized brachyodont generalists whose dental morphocline was roughly parallel to the *Apodemus* stock. The latter phylogenetic entity could then be composed of (b1) the West Palearctic Late Cenozoic (Miocene to Early Pleistocene) representatives of *Micromys* s.l., and (b2) their stem populations supposedly appearing within small-sized forms of *Progonomys-Parapodemus.* These two clades would then compose a panmonophylum, i.e. total taxon covering the crown group of European Pliocene “*Micromys*” and its stem line, distinct from *Apodemus* s.l. and divergent from its East Asiatic sister clade, *Micromys* s.str. In respect to the assumed proximity of *Parapodemus gaudryi*, it seems possible to denote this clade with a generic name *Parapodemus* Schaub, 1938.

**Figure 12:**
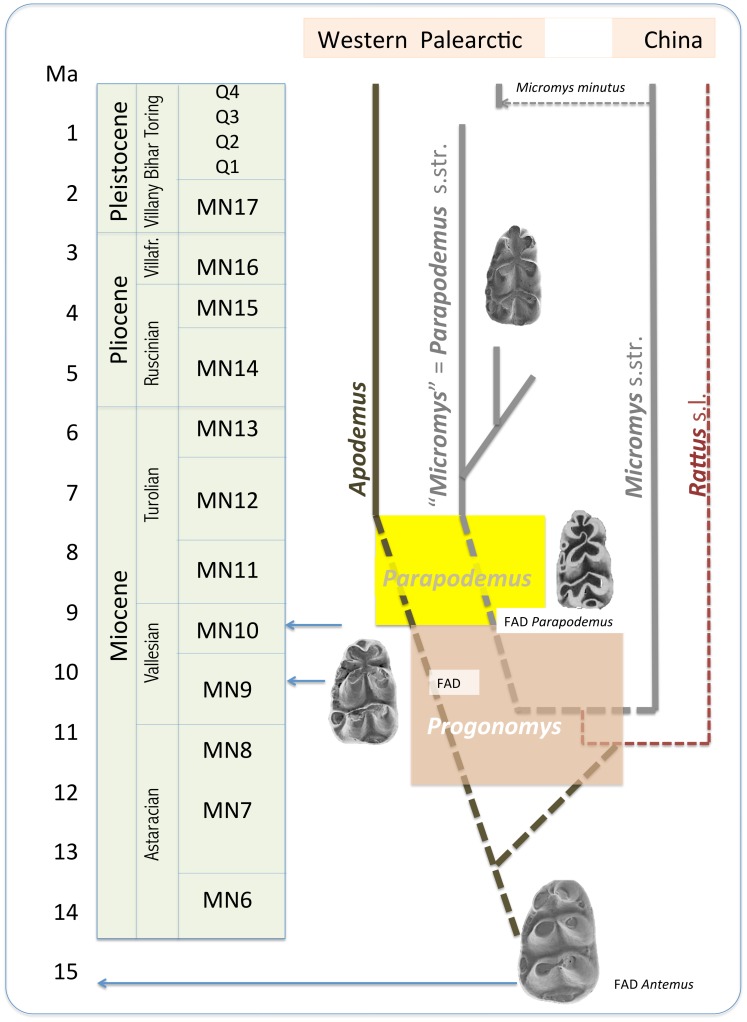
Putative relationship and paleobiogeography of the murine clades discussed in this paper.

Nevertheless, at the same time, it should be emphasized that also the revised view of *Parapodemus* proposed by Martín-Suárez and Mein [56] or Storch [44] remains valid in its essential components. It is undoubtedly well substantiated to consider the large-sized MN11–12 forms (such as *Parapodemus meini* Martín-Suárez et Freudenthal, 2003 and *P. barbarae* van de Weerd, 1976) to be direct ancestors of *Apodemus* s.str., the clade which during MN13–14 diversified into a spectrum of daughter species resembling the current diversity of the genus. Similarly, a continuum of phenotypic transformation from ancestral murids to this clade and its affiliation to the populations formerly arranged under various species of *Parapodemus* also seem to be robustly supported. The exposition of *Apodemus* as a well-defined panmonophylum covering both the greatly diversified Pliocene and Quaternary crown taxa and their stem line, mostly composed of the forms traditionally included in “*Parapodemus*”, provides a robust concept which steadily reminds us that the roots of *Apodemus* history lie in the earliest stages of murid appearance in the Western Palearctic and opens the way to a reliable understanding of the enigmatic past of the group. In the present paper, we attempted the same approach with the West Palearctic early “*Micromys*”, whose appearance is one of the characteristic features of European Mio-Pliocene mammal communities. We identified the roots of this clade among the forms sharing the same phenotypes with the ancestors of *Apodemus* (Fig. 12). Regarding the molecular data on the deep divergence between both clades, the respective phenotypical coalescence suggests unexpectedly broad variation in early members of divergent murid lineages (as Apodemini, Rattini), and almost complete overlap in the patterns of their phenotypic variation. Taken together, these findings reveal a picture characterizing the fossil record of the early evolution of murids more as a firework of phenotypic plasticity than a tree of well stabilized clade-specific morphotypes – indeed, an exciting topic for further study!

### (iii) Nomenclatural Remarks

The nomenclatural consequences of the proposed alternative view on the taxonomic structure of the clade under study are briefly listed below (without discussion of the content or possible relations of the particular named species). Here, it should be remembered that until a profound revision is undertaken this view is merely a working hypothesis.

MURIDAE Illiger, 1811

Rattini Burnbett, 1830

*Micromys* Dehne, 1841

T: *Micromys agilis* Dehne, 1841 = *Mus minutus* Pallas, 1771.

Tentative eidological diagnosis: The East Asiatic clade, small sized forms with weak cingular cusps, minute tma and pronounced tendencies to a reduction in t9 and t12.

Content:

*M.chalceus* Storch, 1987: T MN13 Ertemte 2, China

*M. tedfordi* Wu et Flynn, 1992 (MN15 Yushe, China):

*M.minutus* (Pallas, 1771): The extant form of the panpalearctic distribution, which colonized its current range from its Asiatic center of distribution during the contemporary glacial cycle.

*Parapodemus* Schaub, 1938.

T: *Mus (?Acomys) gaudryi* Dames, 1883 (MN12 Pikermi, Greece).

Tentative eidological diagnosis: The West Palearctic clade, appearing nearly simultaneously with the earliest representatives of the family, i.e. *Progonomys, Apodemus,* differing from them by the slender shape of their molars, the distal position of t1, pronounced crest to cusp-like structures at the palatal base of t8, i.e. presuptive t7, the prolonged anteroconid complex of m1 with well-developed tma, and the appearance of supplementary roots at both m1 and M1.

The clade does *not* include direct ancestors of *Apodemus* such as *P. barbarae* van de Weerd, 1976 or *P. meini* Martín-Suárez et Freudenthal, 1993.

Content:

*? P. lugdunensis* Schaub, 1938 (T: MN11 Mollon, France) (partim)

*P. gaudryi* (Dames, 1883) (MN 12 Pikermi, Greece)

*P. schaubi* Papp, 1947 (T: MN13 Polgardi, Hungary) ? MN14 Podlesice [59]

*P. cingulatus* (Storch et Dahlmann, 1995) (MN13 Maramena, Greece)

*P. paricioi* (Mein, Moissenet et Adrover, 1983) (MN13 Celadas-4B, Spain)

*P. steffensi* (van de Weerd, 1979) (MN14 Kardia, Creece)

*P. bendai* (van de Weerd, 1979) (MN14 Ptolemais 1. Greece)

*P. coronensis* Schaub, 1938 (T: Q2/3 Brasso [41], Schernfeld [60]) = *M. praeminutus* Kretzoi, 1956: MN15 Csarnóta 2, Hungary = *M. caesaris* Minwer-Barakat et al. 2008

*P. kazaniensis* (van de Weerd, 1979) (MN15 Ptolemais 3, Greece).

### Conclusions

*Micromys minutus*, sole species of a remarkable genus of murine rodents (sister clade of rats), is traditionally considered as a taxon with a deep European ancestry, descending from an Early Pliocene form *M. praeminutus*. Yet, molecular phylogeography suggests that *M.minutus* is an E-Asiatic element which invaded the Western Palearctic as late as at ca 80 ky B.P.

To resolve the discrepancy, we reexamined type series of *Micromys praeminutus* and a major bulk of the European Pliocene and Pleistocene records of the genus. We found no essential differences among particular fossil populations but greatly pronounced categorial differences between them and extant *M.minutus* in dental phenotype. The results support the paleobiogeographic scenario proposed by molecular phylogeography and suggest that the European Pliocene and Pleistocene *“Micromys”* represented a specific clade parallel to true *Micromys* whose radiation took place in E-Asia *(chalceus-tedfordi-minutus*). The European clade is then co-identified with a Mio-Pliocene genus *Parapodemus* (which shares several dental apomorphies of the former). Valid name for *M. praeminutus* (covering all European Late Pliocene and Pleistocene forms) is *Parapodemus coronensis* Schaub, 1930.

## Supporting Information

Table S1 **The non-metrical and basic metrical characteristics of studied samples of** ***Micromys*** **in comparison to available literary data.** Part 1: M1 and M2, Part 2: m1 and m2. (MicromysS2table.xls file)
(XLSX)

Dataset S1 **List of the European fossil records of** ***Micromys.***
(DOCX)
