# Supplementary material for: Late Cenozoic History of the Genus Micromys (Mammalia, Rodentia) in Central Europe
Source: PLoS One. 2013 May 6;8(5):e62498. doi: 10.1371/journal.pone.0062498 (PMC3646007; doi:10.1371/journal.pone.0062498)
Supplement: Dataset S1 — List of the European fossil records of Micromys. (DOCX) [file pone.0062498.s002.docx]

**Supporting information**

**Dataset S1**: **List of the European fossil records of *Micromys***

Arranged by stratigraphic units. The sites providing more than 10 items are indicated by asterisk (*), the material examined in frame of this paper in **bold**.

MN13- MN14

Rema Marmara, Greece ([61]-de Bruijn 1989, [62]-Koufos 2006: *Micromys* sp.), Celadas 4, Teruel, Spain ([7]-Mein et al. 1983: type locality of *M. paricioi*), Peralejos E, Spain ([7]-Mein et al. 1983: *M. paricioi),* MN13 Granada Basis MNA-2, DHS-16, CAC-5,11 ([37]-García-Alix et al. 2008: *M. paricioi),* *Maramena, Greece ([9]-Storch and Dahlmann 1995: type locality of *M. cingulatus*), Silata, Greece ([63])-Vasileaiadou et al. 2003: *M.*cf. *paricioi*), Kessani, Greece ([64]-Vasileaiadou et al. 2012: *M. steffensi*), *Igdeli, Turkey ([65]-Suata Alpaslan et al. 2010: *M. bendai*), Vinogradovka 1, Ukraine ([23]-Nesin snd Storch 2004: *M. cingulatus* and *M. chalceus*).

MN15

***Csarnóta 2**, Hungary ([11]-Kretzoi 1959: type locality of *M. praeminutus*), *Węże, Poland ([39]-Sulimski 1964: *M. praeminutus),* **Gundersheim-Findling**, Germany ([22]-Fejfar and Storch 1990: *M. praeminutus*), * Ptolemais 1, Greece ([8]-van de Weerd 1979: type locality of *M. bendai*), * Ptolemais 3, Greece ([8]-van de Weerd 1979: type locality of *M. kozaniensis*), *Kardia, Greece ([8]-van de Weerd 1979: type locality of *M. steffensi),* Sète, France ([12]-Michaux 1969: *M. praeminutus* ), Saze, France ([66]-Michaux 1971: *M. praeminutus* ), Moreda 2, Spain ([8]-van de Weerd 1979: *M. praeminutus* ), Limni 5, Greece ([8]-van de Weerd 1979: *M. bendai*), Limni 6, Greece ([8]-van de Weerd 1979: *M. praeminutus*), **Beremend 11**, Hungary ([67]-Jánossy 1986: *M. praeminutus* ), **Vinogradovka 2, 3,** Ukraine ([23]-Nesin and Storch 2004: *M. praeminutus*), Obukhovka 2 ([23]-Nesin and Storch 2004: *M. praeminutus*).

MN16

**Ręmbielice Królewskie I, II**, Poland ([24]-Kowalski 1960: *M. praeminutus*),*Toll de Chiclana 13(TCH 13), Gaudix Basin, Spain ([68], [10]-Minwer-Barakat et al. 2005, 2008: type locality of *M. caesaris)* **Zhevakhova Gora 15** ([23]-Nesin and Storch 2004: *Microtus praeminutus*), **Gundersheim**, Germany (unidentified item in original collection, not mentioned by [25]-Heller 1936), Podari, Romania ([15]-Kowalski 2001: *Micromys praeminutus*).

MN17

Valdeganga II, III, IV, Spain ([7]-Mein et al. 1983: *M*. aff. *minutus,* coidentified with *M. caesaris* by [10]-Minwer-Barakat et al. 2008 based on direct comparison), * **Včeláre 6/1,** Slovakia ([19]-Horáček 1985: *M. praeminutus* ), *Mas Rambault 2, France ([38]-Aguilar et al. 2002: *M*. cf. *praeminutus),* Zuurland 91-92m, Netherlands ([14]-Reumer 2003: *M. praeminutus*), , **Kotlovina 3**, Ukraine ([23]-Nesin and Storch 2004: *M. praeminutus*), **Zamkova Dolna Cave**, Poland ([27]-Nadachowski, 1990: *Micromys* not included in the list), Osztramos 3 ([67]-Jánossy 1986: *M.* cf. *praeminutus*).

Q1

Zuurland 42-43m, Netherlands ([14]-Reumer 2003: *M. minutus*), **Včeláre 6/3**, Slovakia ([19]-Horáček 1985: *Micromys* cf. *praeminutus*), Bol’shevik 2, Ukraine ([69]-Rekovets 1994, [16]-Krochmal and Rekovets 2010: *Micromys* sp.), Zeli, Greece ([70]-Meulen and Kolfschoten 1986: *M.* cf. *minutus*)

Q2

**Hohensülzen,** Germany ([13]-Storch et al. 1973: *Micromys minutus*), Huéscar 1, Spain ([71]-Agustí et al. 2010: *M. minutus*), Gran Dolina TD5, TD 6 ([18]-López Antonanzas and Cuenca Béscos 2002: *M. minutus*)

Q3

Medzhibozh 1, Ukraine ([69]-Rekovets 1994, [16]-Krochmal and Rekovets 2010: *Micromys* sp.), Bol’shaja Kmyshevakha, Ukraine ([69]-Rekovets 1994, [16]-Krochmal and Rekovets 2010: *Micromys* sp*.*), Ozernoje I, Ukraine ([69]-Rekovets 1994, [16]-Krochmal and Rekovets 2010: *M. minutus*), Arago, France ([15]-Kowalski 2001: *M.* cf. *minutus*), Vaufrey, France ([72]-Marquet 1993: *M. minutus*)

Q4

**Tarkö – Vistualian layer**, Hungary ([73]-Jánossy 1976: *M. minutus*), Raspopintsy, Ukraine ([69]-Rekovets 1994, [16]-Krochmal and Rekovets 2010: *M. minutus*), **Soutěska II, Zazděná, Bašta,** all Czech Republic (see [20]-Horáček and Ložek 1988 for details), Piesede bei Malchin ([74]-Heinrich and Maul 1983: *M. minutus*), La grotte de la Chauve-Souris a Donzere (Drome, France) ([75]-Jeannet and Vital 2009: *M. minutus* )
